# Electricity in Alveolar Inflammations

**Published:** 1897-06

**Authors:** William Rollins


### ELECTRICITY IN ALVEOLAR INFLAMMATIONS.

BY WILLIAM ROLLINS.

In this journal I have called attention to the use of high-voltage continuous currents in treating inflammations about the jaws, and described a convenient form of generator. While testing this form of current I have also built a number of generators for producing the Tesla or high-frequency current, constructing them in accordance with the ideas of Tesla, Hertz, and Lodge, and as this form of current seems to be equally efficient and more certainly generated, a brief description of a very simple form of apparatus will be here described, which, though it contains no new ideas, is convenient in practical use, and may help some one who wishes to experiment in this direction.

First, the position of the resistance is of importance. On each main of the one hundred-and-ten-volt Edison street circuit I place a carbon resistance of a sufficient number of ohms to allow one, two, three, or four ampères of current to pass. This resistance is placed on the wall, out of reach of danger from short circuit, which, as in using this current in cataphoresis, is to be carefully guarded against.

The wires from this permanent resistance are brought to another variable resistance near the operator's chair. The wires leading from this second resistance are connected with the terminals of the primary of an induction coil, in the circuit of which is a rapid break. The terminals of the secondary of this coil lead to the primary of a second coil with the usual spark gap. In the secondary of this coil the patient is placed. Enough current may be passed through his body to feebly excite a Crookes tube in series with him without pain. Tesla showed some years ago that an ampèrage great enough to produce serious consequences, with a slow interruption, was harmless and painless if the interruption were rapid, so that no one need hesitate to use such a current in dentistry, if arranged as here described, provided the patient takes hold of the terminals before the current starts and holds them until it stops. The mechanism for stopping the current is a foot-switch, which is placed in the main circuit, between the patient and the resistances. If this matter proves of sufficient interest, I shall be glad to figure more perfect, though more complicated, apparatus for the purpose.

